# Repeated Exposure of *Escherichia coli* to High Ciprofloxacin Concentrations Selects *gyrB* Mutants That Show Fluoroquinolone-Specific Hyperpersistence

**DOI:** 10.3389/fmicb.2022.908296

**Published:** 2022-05-30

**Authors:** Aurore Perault, Catherine Turlan, Nathalie Eynard, Quentin Vallé, Alain Bousquet-Mélou, Etienne Giraud

**Affiliations:** ^1^INTHERES, Université de Toulouse, INRAE, ENVT, Toulouse, France; ^2^Service d’Ingénierie Génétique du LMGM (SIG-LMGM-CBI), CNRS, Toulouse, France

**Keywords:** experimental evolution, DNA gyrase, tolerance, persisters, quinolones

## Abstract

Recent studies have shown that not only resistance, but also tolerance/persistence levels can evolve rapidly in bacteria exposed to repeated antibiotic treatments. We used *in vitro* evolution to assess whether tolerant/hyperpersistent *Escherichia coli* ATCC25922 mutants could be selected under repeated exposure to a high ciprofloxacin concentration. Among two out of three independent evolution lines, we observed the emergence of *gyrB* mutants showing an hyperpersistence phenotype specific to fluoroquinolones, but no significant MIC increase. The identified mutation gives rise to a L422P substitution in GyrB, that is, outside of the canonical GyrB QRDR. Our results indicate that mutations in overlooked regions of quinolone target genes may impair the efficacy of treatments *via* an increase of persistence rather than resistance level, and support the idea that, in addition to resistance, phenotypes of tolerance/persistence of infectious bacterial strains should receive considerations in the choice of antibiotic therapies.

## Introduction

Since the discovery of the antibacterial activity of penicillin (Fleming, 1929), antibiotics have been used to treat bacterial infections. However, their overuse and misuse over the decades have contributed to the development in many bacterial pathogens of strategies allowing them to overcome antibiotic treatments. In addition to antibiotic resistance, which has been extensively studied, tolerance and persistence mechanisms are now increasingly looked at as important factors of antibiotic treatments failures (Windels et al., 2019, 2020).

Resistance is the inherited ability of bacteria to grow in the presence of therapeutic concentrations of antibiotics, regardless of the duration of treatment (Brauner et al., 2016). Resistance levels can be quantified by several *in vitro* methods, one of them being the determination of the Minimum Inhibitory Concentration (MIC; Balaban et al., 2019), that is, the minimal antibiotic concentration that prevents growth of a bacterial population for a specific antibiotic. MICs are routinely determined in clinical settings and are crucial for antibiotic, dosing, and regimen selection. However, resistance is not the only strategy used by bacteria to survive antibiotic treatments.

Tolerance is defined as the ability of a bacterial population to transiently survive high antibiotic concentrations without an increase in the MIC (Balaban et al., 2019). Experimental *in vitro* studies of bacterial evolution have shown the existence of tolerance mutations resulting in a slower killing of the whole bacterial population (Balaban et al., 2019). Tolerance has long been considered as a simple phenotypic adaptation allowing bacteria to prevent total eradication under stress conditions. However it has been highlighted that increased tolerance levels can be conferred by chromosomal mutations in genes involved in global metabolism or stress regulation (Van den Bergh et al., 2017; Chebotar’ et al., 2021). For example, mutations have been identified in genes involved in activation of the stringent response and thus in the inhibition of bacterial growth, such as genes encoding the *hipAB* toxin–antitoxin module (Korch et al., 2003) or the methionine-tRNA ligase in *Escherichia coli* (Girgis et al., 2012; Levin-Reisman et al., 2017). Importantly, these mutations subsequently promote the secondary selection of resistance mutations (Van den Bergh et al., 2016; Levin-Reisman et al., 2017).

Another phenomenon called persistence has been defined in a recent consensus statement, as the ability of a subset of bacteria, called persisters, to survive a transient exposure to a bactericidal antibiotic concentration (Balaban et al., 2019). Persisters are stochastically produced drug-tolerant cells. They are phenotypic variants that do not grow and are killed much more slowly than the major antibiotic-susceptible bacterial population, which typically results in biphasic killing curves when performing time-kill assays (Brauner et al., 2016; Balaban et al., 2019). The dormancy state of persisters cells is considered to be responsible for their high tolerance to antibiotics. Upon removal of antibiotics, persisters can resume growth. This makes them a recognized cause of recalcitrance of bacterial infections to antibiotic treatments. Bacterial strains that produce increased persisters subpopulations are termed high persistence or hyperpersistent strains. Experimental *in vitro* studies of bacterial evolution have shown the existence of high persistence mutations that increase the persisters fraction (Khare and Tavazoie, 2020; Sulaiman and Lam, 2020). Recent laboratory evolution experiments have shown that repeated exposure of an *E. coli* strain to high antibiotic concentrations including fluoroquinolones can select hyperpersistent mutants with mutations in translation-related genes (Khare and Tavazoie, 2020; Sulaiman and Lam, 2020).

Tolerance and persistence phenomena can be evidenced by time-kill measurements and quantified by MDK (“Minimum Duration for Killing”), which is the minimum time required to kill a certain percentage of the bacterial population. For example, MDK_99_ and MDK_99.99_ are the minimum duration to kill 99 and 99.99% of the bacterial population, respectively (Brauner et al., 2016).

In this context, we wondered whether tolerance or hyperpersistence mutations could be selected by repeated exposure of a wild-type susceptible *E. coli* strain to clinically relevant concentrations of ciprofloxacin, an antibiotic which targets type II topoisomerases, and primarily DNA gyrase (Vance-Bryan et al., 1990). An Adaptative Laboratory Evolution (ALE) experiment was therefore conducted and evolved clones showing an increased survival during antibiotic treatment or resistance were phenotypically and genotypically analyzed. A summary diagram is presented at Supplementary Figure S1. Our results showed that *gyrB* mutations conferring a fluoroquinolone-specific hyperpersistent phenotype, rather than a classical resistance phenotype, could be selected under repeated exposition to clinically relevant ciprofloxacin concentrations.

## Materials and Methods

### Bacterial Strains and Culture Conditions

We used *E. coli* ATCC25922-GFP-Amp^R^, named H1 thereafter as the parental strain in our evolution experiments. All strains were grown in Mueller–Hinton Broth (MHB) at 37°C, except during site-directed mutagenesis experiments, where Luria-Bertani Broth (LB) was used. Three different strains of *E. coli* (*E. coli* ATCC25922, *E. coli* BL21, and *E. coli* MG1655) were used in site-directed mutagenesis. For bacterial counts in persistence assays, we used tryptic soy agar supplemented with magnesium heptahydrate sulfate and activated charcoal.

### Adaptative Laboratory Evolution Experiment

**Figure 1:**
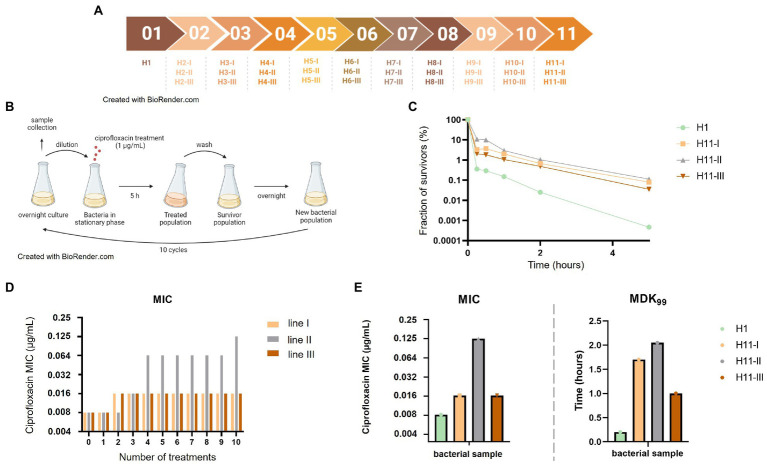
Repeated exposures to ciprofloxacin lead to the emergence of hyperpersistence and resistance phenotypes. **(A)** Chronological set up of the different antibiotic treatments and bacterial population samples evolved from the H1 parental strain. **(B)** Set up of the evolutionary experience with repeated ciprofloxacin treatments. Bacteria were exposed to 10 ciprofloxacin treatments as follows: bacteria were grown overnight, stationary phase cultures were sampled and diluted by a factor of 1:30 before addition of ciprofloxacin at 1 μg/ml for 5 h. After antibiotic removal by centrifugation and washing, all bacteria were grown again overnight in fresh medium for the next cycle. **(C)** Time-kill assays performed on bacterial populations obtained after 10 ciprofloxacin treatments. **(D)** Evolution of ciprofloxacin MIC of bacterial populations along treatments. **(E)** Evolved bacteria from the three evolution lines showed increased MDK_99_.

Three independent evolution lines (named I, II, and III thereafter) were conducted in parallel, starting from overnight cultures of the parental *E. coli* ATCC25922-GFP-Amp^R^ strain in MHB supplemented with 50 μg/ml ampicillin. A total of 10 selection cycles were performed for each line (Figures 1A,B) as follows. At each cycle, 1 ml of overnight cultures grown from the previous cycle were transferred to 29 ml of MHB (1:30 dilution) in Erlenmeyer flasks. Bacteria were enumerated and, ciprofloxacin was then added at a final concentration of 1 μg/ml. Cultures were then incubated for 5 h at 37°C with agitation at 180 rpm. This ciprofloxacin concentration far above the MIC of the parental strain (0.008 μg/ml) was used to prevent the probability of selecting resistant bacteria and rather promote the selection of tolerant or hyperpersistent mutants. Cells from the treated cultures were collected by centrifugation, washed to remove ciprofloxacin, and resuspended in 10 ml of MHB. Ten-fold serial dilutions were prepared to enumerate surviving bacteria. Cultures were then incubated overnight at 37°C with ampicillin at 50 μg/ml, and newly grown bacteria were used for the next selection cycle. Samples of bacterial populations from all overnight cultures were conserved at −80°C.

### Selection of a Resistant *gyrB* Mutant

A spontaneous resistant mutant “R” was selected by plating cultures of the *E. coli* ATCC25922-GFP-Amp^R^ strain on MH agar medium supplemented with ciprofloxacin at 4-fold the parental MIC.

### Susceptibility Testing

Minimum Inhibitory Concentration determinations were performed on evolving bacterial populations and on isolated clones according to the microdilution method (Andrews, 2001). The MIC was determined as the lowest antibiotic concentration where no bacterial growth occurred. Results were validated by comparison with reference values provided by EUCAST for the *E. coli* ATCC25922 strain (The European Committee on Antimicrobial Susceptibility Testing, 2022).

### Persistence Assays

Overnight cultures were first diluted 1:6 or 1:600 in fresh MHB for 10^7^–10^8^ CFU/ml inocula and 10^5^–10^6^ CFU/ml inocula, respectively. Initial CFU counts were determined by serial dilution and plating as described above. To overcome resistance phenotypes, time-kill assays were performed at antibiotic concentrations much higher than the MICs (128- or 256-fold higher) for each strain. Antibiotic used were ciprofloxacin, enrofloxacin, norfloxacin, nalidixic acid, flumequine, tetracycline, and gentamicin (Sigma and Fluka). The survival fraction was calculated as the ratio of bacterial numeration at a given time of the treatment versus the bacterial numeration before treatment.

### Whole-Genome Sequencing and Identification of Mutations

Genomic DNA was extracted using the DNEasy Blood and Tissue kit (QIAGEN). A whole-genome sequencing (WGS) was performed using IonTorrent technology at the GeT-PlaGe Genotoul Platform, Toulouse, France. Sequence assembly was performed, and mutations were identified by comparison with the *E. coli* ATCC25922 reference genome (GenBank accession number: CP009072).

### Strain Reconstruction by Site-Directed Mutagenesis

Mutagenesis was based on the one-step inactivation of chromosomal gene strategy (Datsenko and Wanner, 2000). The different recombination substrates were obtained by PCR amplification and assembly. Primers used for this directed mutagenesis are described in Supplementary Table S1. The plasmid pKD3 was used as template to amplify the FRT-flanked chloramphenicol resistance (cat) gene, antibiotic that will be used later for the selection of recombinant clones. PCR reaction was performed using the PrimeSTAR® Max DNA Polymerase (Takara). DNA fragments isolated from agarose gels were purified using the GFX PCR DNA and gel band purification kit (Sigma Aldrich). For this mutagenesis, we used plasmids pKD46 and pE-FLP containing an ampicillin resistance gene and a heat-sensitive replication origin, and from which λRed proteins or FLP are constitutively produced.

Fifty microliters of electrocompetent cells were mixed with 500 ng of recombinant substrates or 50 ng of plasmid and incubated for 20 min in ice. Electroporation was performed at 2.5 kV, 25 μF, 200 Ω during 4.5 ms into a chilled 2 mm cuvette 1 ml of SOC has been added, and cells recovery was done at 30°C or 37°C for 2 h. Cells were plated on LB Ampicillin or LB Chloramphenicol and incubated at 30°C or 37°C.

To verify the introduction of the *gyrB* gene mutations, PCR sequencing was performed from an overnight culture diluted at 1:13 and preincubated at 95°C 5 min, using the PrimerSTAR® Max DNA Polymerase (Takara) with the RecF5 and RecF6 primers (Supplementary Table S1). This amplification produced a 1,070 bp fragment which was then sequenced.

To eliminate the chloramphenicol resistance gene, Cm^R^ mutants were transformed with pE-FLP plasmid, as previously described (Datsenko and Wanner, 2000) and ampicillin-resistant transformants were selected at 30°C. Transformant clones were streaked and incubated at 42°C on non-selective media and then tested for chloramphenicol sensitivity. Excision of the *cat* gene was verified by PCR using the Taq DNA polymerase (MP Biomedical) with the RecF5 and RecF14 primers (Supplementary Table S1) and sequencing from RecF13.

### Statistical Analysis

All statistical analyses were performed using data from independent biological replicates. Differences between groups of quantitative variables were assessed using the Kruskal–Wallis test. Differences were considered significant when *p*-values were ≤0.05.

## Results

### After 10 Days of Ciprofloxacin Treatment, Altered Bactericidal Activity Is Observed With Increased Persisters Fraction and MDK Levels

An experimental evolution experiment was set up in order to select tolerant or hyperpersistent bacteria from a susceptible parental strain (Figure 1A). The fully quinolone-susceptible *E. coli* ATCC25922-GFP strain, which was used as the parental strain H1, was repeatedly exposed to 1 μg/ml ciprofloxacin, a concentration 128-fold higher than its MIC (0.008 μg/ml). Ten selection cycles were performed along 15 days, each cycle consisting in the ciprofloxacin treatment of a stationary phase culture obtained from growth of bacteria that had survived the previous treatment. Three independent evolution lines were conducted in parallel (Figures 1A,B). To evaluate how resistance and tolerance evolved in the three lines, samples of bacterial populations were collected at each cycle and their ciprofloxacin MICs and survival fractions under ciprofloxacin treatment were determined.

For unevolved population directly grown from the parental strain (H1), the ciprofloxacin MIC was 0.008 μg/ml and the survival fraction after a 5-h exposure to 1 μg/ml ciprofloxacin was in the 5.10^−5^–10^−6^ range (Figure 1C). In lines I and III, the ciprofloxacin MIC of bacterial populations increased 2-fold after two treatments but did not change thereafter (Figure 1D). In contrast, the ciprofloxacin MIC of the bacterial population of line II increased 16-fold along the 10 treatments, to reach the value of 0.125 μg/ml.

Time-kill assays were performed on bacterial populations evolved along 10 treatments (H11-I, H11-II, and H11-III; Figure 1C), and MDK_99_ values were deduced from killing curves. Bacterial populations from all three evolutionary lines showed similarly altered killing, with a 100-fold increased persisters fraction after a 5-h ciprofloxacin treatment, compared to the parental strain they derive from.

MDK_99_ values of evolved bacterial populations increased 5- to 10-fold for all three lines (Figure 1E). For H11-I and H11-III (i.e., evolved populations from lines I and III, respectively), the increase in survival fraction and MDK_99_ was associated with a ciprofloxacin MIC similar (only 2-fold higher) to that of the parental strain, indicating that the altered killing was due to a tolerance or hyperpersistence mechanism. In contrast, increased survival and MDK_99_ observed for population H11-II was associated to a significantly increased ciprofloxacin MIC (16-fold higher than the MIC of the H1 parental strain), indicating that the killing in bacteria evolved in this line was altered probably due to a higher level of resistance.

In summary, it appeared that increased survival observed for the bacterial populations of the three evolutions lines after 10 treatments were due to a tolerant or hyperpersistent phenotype in line I and III, but rather to a resistance mechanism in line II. This was confirmed by analysis of individual clones isolated from populations of the three evolution lines. Table 1 shows resistance and persistence characteristics of representative clones.

**Table 1:** Mutations identified in resistant and hyperpersistent mutants and their phenotypic characteristics in the presence of ciprofloxacin.

Strain	Identified mutations	Gene	Ciprofloxacin MICs (μg/ml)	Ciprofloxacin MDK_99_ (h)	Ciprofloxacin MDK_99.99_ (h)	Survival fraction at 24 h
H1	/	/	0.008	0.4	4.2	2.5 × 10^−7^
Resistant R	S464Y (TCT → TAT)	*gyrB*	0.062	2.1	7.6	3.6 × 10^−7^
H11-l1	L422P (CTG → TTG)	*gyrB*	0.016	16	>24	3.8 × 10^−3^
H11-III2	L422P (CTG → TTG)	*gyrB*	0.016	1.6	9.5	2.6 × 10^−5^
	T insertion at nucleotide position 2093745	MFS family				
H11-II4	D87G (GAC → GGC)	*gyrA*	0.125	1.6	6.6	1.1 × 10^−5^

Survival fraction was determined after exposing bacteria for 24 h to a ciprofloxacin concentration of 256 times their MIC. The survival fraction was calculated by dividing the number of surviving bacteria by the number of live bacteria that were present before the antibiotic treatment. The nucleotide position refers to the *Escherichia coli* ATCC25922 genome (GenBank accession number CP009072).

### The GyrB L422P Substitution Is Associated to an Hyperpersistence Phenotype

To identify the mutations responsible for the previously identified hyperpersistence and resistance phenotypes, whole-genome sequencing was performed on clones isolated from the different lines. In addition, for comparison purpose, a spontaneous resistant mutant (named R) obtained by plating a culture of the parental strain on a ciprofloxacin-supplemented solid medium, was also sequenced. Interestingly, all spontaneous and evolved mutants had a mutation on one of the two subunits of the DNA gyrase, which is the enzyme primarily targeted by quinolones (Table 1).

The R mutant (ciprofloxacin MIC: 0.062 μg/ml) showed a mutation on the *gyrB* gene giving rise to the S464Y amino acid change. Mutations at this position are known to confer a quinolone resistance phenotype. This substitution has already been described in other Gram-negative species, such as *Salmonella* spp. (Correia et al., 2017), *Pseudomonas aeruginosa* (Mouneimné et al., 1999), and *Proteus mirabilis* (Weigel et al., 2002).

Clone H11-II4 isolated from evolution line II, showing a resistance phenotype, carried a mutation on the *gyrA* gene giving rise to the D87G amino acid substitution. This substitution is frequent in fluoroquinolone-resistant gram-negative bacteria and particularly in enterobacterales (Conrad et al., 1996; Correia et al., 2017).

More interestingly, three sequenced clones from line I (H11-I1, H11-I3, and H11-I5) and clone H11-III2 from line III carried the same mutation of the *gyrB* gene giving rise to the amino acid change L422P. In clone H11-III2, an additional mutation consisting in a single nucleotide insertion was also identified in a major facilitator superfamily (MFS) gene (Table 1). This frameshift mutation probably causes a loss of function of the corresponding MFS protein.

The L422P substitution was associated in our isolates with a small (2-fold) increase of ciprofloxacin MIC, but with a high persistence level. From there, we focused our investigations on isolate H11-I1, as representative of the hyperpersistence-associated genotype. This isolate displays a MDK_99_ increased 40-fold and a survival fraction after a 24-h treatment increased more than 10^4^-fold, compared to its parental strain.

### The Hyperpersistent Phenotype Associated With the Mutation L422P Is Specific to Fluoroquinolones

Identification of mutations in the DNA gyrase genes suggested that the phenotypes selected in our experiments were specific to quinolones. Therefore, we investigated whether experimental selections for tolerance/persistence and resistance to ciprofloxacin also resulted in persistence and resistance to other quinolone antibiotics.

**Figure 2:**
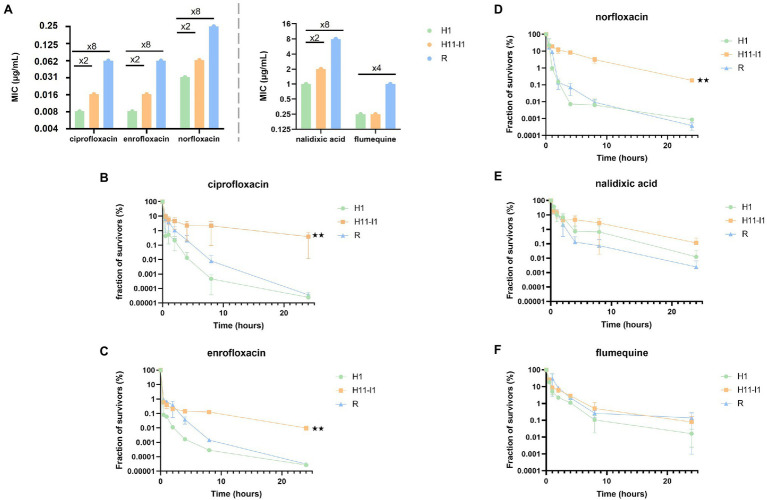
The L422P substitution in GyrB is associated with an hyperpersistence phenotype specific to fluoroquinolones. **(A)** MICs of the parental strain (H1), of the H11-I1 mutant clone from line I and of a spontaneous resistant mutant (R) were determined for three fluoroquinolones (ciprofloxacin, norfloxacin, and enrofloxacin) and two older generation quinolones (nalidixic acid and flumequine). **(B–D)** Bactericidal assays in the presence of fluoroquinolones at 256-fold the MIC of each strain (ciprofloxacin, enrofloxacin, and norfloxacin) showed an altered bactericidal effect for the H11-I1 mutant, with a fraction of persisters increased by a factor 100 to 1,000. **(E,F)** Bactericidal essays in the presence of old generation quinolones (nalidixic acid and flumequine) showed no significant alteration of bactericidal effect for the H11-I1 mutant. Significant *p*-values compared with the H1 strain are noted (^**^p ≤ 0.01).

The MIC of three fluoroquinolones (ciprofloxacin, enrofloxacin, norfloxacin) and of two quinolones (flumequine, nalidixic acid) were measured for the susceptible (H1), hyperpersistent (H11-I1) and resistant (R) strains (Figure 2A). The susceptibility level of the hyperpersistent strain for these five antibiotics was similar (unchanged or only doubled MICs) to that of the parental H1 strain. In contrast, the MICs of the resistant strain showed an 8-fold increase for ciprofloxacin, norfloxacin, enrofloxacin, and nalidixic acid and 4-fold for flumequine.

Tolerance and persistence levels were also measured for the five antibiotics (Figures 2B–F). All killing curves showed the same biphasic profile characteristic of a persistence phenotype, that is, presence in bacterial populations of persisters tolerant to the tested antibiotics. The bactericidal activity of ciprofloxacin, norfloxacin, and enrofloxacin against the hyperpersistent H11-I1 strain was significantly decreased compared to that observed against the H1 parental and the resistant strains (Figures 2B–D). This resulted in increased MDK_99_, MDK_99.99,_ and 24-h survival rates (Supplementary Table S2). Interestingly, results were different for flumequine and nalidixic acid, which are not fluoroquinolones. For these two older generation quinolones, bactericidal profiles observed for resistant and tolerant mutant were not significantly different from those obtained for the susceptible parental strain (Figures 2E,F).

Effect of the inoculum size was also analyzed, since many studies have shown that it can influence killing profiles during treatments with fluoroquinolones (Li et al., 2017). An inoculum of 10^5^ bacteria was therefore exposed to the same concentrations of ciprofloxacin as in previously described killing assays. As with the large size inoculum, a bimodal killing pattern was observed with a significantly higher persisters rate for the H11-I1 strain compared to its parental strain (H1; Supplementary Figure S2). This shows that the hyperpersistent phenotype of this mutant is not dependent on inoculum size.

These results show that the hyperpersistent mutant H11-I1, despite displaying no significant increase in resistance, is killed much less efficiently by fluoroquinolones than its susceptible parental strain and than a resistant mutant. This hyperpersistence phenotype appears to be specific to fluoroquinolones since it is not observed for flumequine and nalidixic acid.

In a second step, we addressed whether experimental selections of tolerance and resistance to ciprofloxacin also resulted in cross-persistence and cross-resistance to antibiotic of other families.

For the three strains studied, parental (H1), hyperpersistent (H11-I1), and resistant (R), MICs of tetracycline and of gentamicin (aminoglycoside family) were measured. Susceptibility levels of the hyperpersistent and resistant mutant strains to these two antibiotics appeared identical to that of the parental H1 strain (Supplementary Figure S3A).

Tolerance/persistence levels of the susceptible, hyperpersistent, and resistant strains were then measured for the two antibiotics. No significant difference in the bactericidal activity of gentamicin and tetracycline against the resistant and hyperpersistent strains was observed compared to the susceptible strain (Supplementary Figures S3B,C). No difference in MDK_99_ and MDK_99.99_ was observed between the three strains when treated with gentamicin. Nevertheless, the MDK_99_ of the H11-I1 mutant was increased about 2-fold during tetracycline treatment compared to those of the parental and resistant strains (Supplementary Figure S3D). In summary, the hyperpersistence phenotype of the H11-I1 mutant seems to be associated with fluoroquinolones only and no cross-persistence is observed.

### Induction of the Hyperpersistence Phenotype in the Presence of the GyrB Mutation L422P Is Strain-Dependent

In order to confirm that the GyrB L422P substitution was causal to the hyperpersistence phenotype of H11-I1 mutant and test the influence of the genetic background, the mutation was introduced by directed mutagenesis in 3 *E. coli* strains (*E. coli* ATCC25922, MG1655, and BL21). As a control, the resistance mutation identified in the R mutant was also reconstructed in the three genetic backgrounds. All strains were then submitted to MIC determinations and time-kill assays.

**Figure 3:**
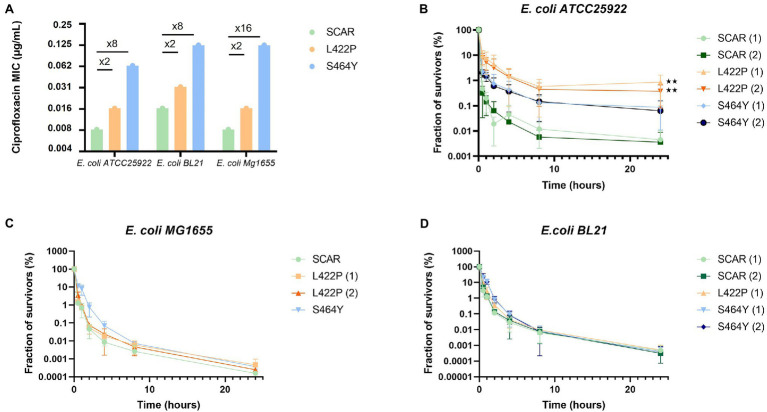
The hyperpersistent phenotype associated with the GyrB L422P substitution is strain-dependent. **(A)** Levels of resistance to ciprofloxacin of reconstructed mutants of *Escherichia coli* ATCC25922, BL21, and MG1655 carrying the GyrB L422P or S464Y substitutions. SCAR strains are susceptible controls with no introduced mutation in *gyrB*, but containing the scar left by removal of the chloramphenicol resistance gene used for selection of recombinant clones. For each mutation, when possible, two independent recombinant clones were analyzed and then named (1) and (2). MICs of the three strains of *E. coli* with the L422P substitution were similar to those of the susceptible strains (SCAR) with only a 2-fold factor difference. **(B)** Bactericidal assays performed on the reconstructed *E. coli* ATCC25922 strains showed an alteration in bactericidal activity in the presence of ciprofloxacin for *E. coli* ATCC25922 (L422P) clones. **(C,D)** Bactericidal assays conducted on strains *E. coli* MG1655 and BL21 (L422P), (S464Y), and (SCAR) showed no alteration in bactericidal activity in the presence of ciprofloxacin. Significant *p*-values compared to SCAR1 and SCAR2 control clones are noted (^**^p ≤ 0.01).

In the reconstructed strains, introduction of the GyrB L422P and S464Y substitutions resulted in ciprofloxacin MICs increases similar to those observed in the mutants selected during the experimental evolution, that is, a 2-fold increase for the L422P substitution and a 8- to 16-fold increase for the S464Y substitution (Figure 3A).

As expected, the survival fraction at 24 h of the *E. coli* ATCC25922 (L422P) reconstructed mutant was increased 100-fold, confirming the causality of L422P substitution to the phenotype of hyperpersistent to ciprofloxacin (Figure 3B). Killing assays also conducted on *E. coli* ATCC25922 (L422P) with other fluoroquinolones, older quinolones, an aminoglycoside, and tetracycline gave results similar to those observed with the strains selected during ALE experiments. These results confirmed that L422P substitution conferred an hyperpersistent phenotype specific to fluoroquinolones (Supplementary Figure S4).

In contrast, no alteration of bacterial killing was observed when bacteria were treated with fluoroquinolones after introduction of the L422P substitution in *E. coli* MG1655 and BL21 strains (Figures 3C,D). This indicates that expression of hyperpersistence phenotype associated to GyrB L422P substitution does not occur in all genetic backgrounds but is actually strain-dependent.

## Discussion

Antibiotic tolerance and hyperpersistence have been associated to chronic and recurrent infections (Lewis, 2010, 2019; Van den Bergh et al., 2017). In particular, tolerance and hyperpersistence mechanisms are suspected to be involved in the recurrency of Urinary Track Infections (UTI; Murray et al., 2021). As these infections are often caused by *E. coli* and may be treated with ciprofloxacin, we designed this study to address whether repeated *in vitro* exposure of an *E. coli* strain to high concentrations of ciprofloxacin could select tolerant or hyperpersistent clones. Study of such phenotypes is important since previous studies have shown that selection of antibiotic resistance, which is the main cause of recalcitrance to antibiotic therapy, is more likely to occur in tolerant or hyperpersistent strains (Levin-Reisman et al., 2017).

Starting from a susceptible *E. coli* strain, we observed the selection of bacterial clones displaying a significantly higher persistence rate in time-kill assays carried out with ciprofloxacin, but a resistance similar to their parental strain. Interestingly, we discovered that this hyperpersistence phenotype was associated with a mutation in the *gyrB* gene coding for the B subunit of DNA gyrase, which is the main target enzyme of quinolones. This result was unexpected since mutations in quinolone target genes usually confer increased resistance to antibiotics of this family (Hooper and Jacoby, 2015). In contrast, tolerance/persistence mechanisms are generally associated with mutations located outside the antibiotic target genes, in global metabolism or stress regulation genes (Van den Bergh et al., 2017; Chebotar’ et al., 2021). Quinolone resistance mutations appear at specific codons of regions called QRDR (Quinolone Resistance Determining Region). They are more frequent in the *gyrA* QRDR (spanning codons 64–106) than in the *gyrB* QRDR (codons 426–464; Bhatnagar and Wong, 2019). The mutation identified in our hyperpersistent mutant, which gives rise to the L422P amino acid substitution, is therefore outside but close to the *E. coli gyrB* QRDR. Interestingly, the 422 amino acid position is located into the TOPRIM domain which is the catalytic domain involved in DNA strand breakage and rejoining (Bhatnagar and Wong, 2019). The presence of this mutation in a quinolone target gene suggested that the hyperpersistence phenotype could be specific to these antibiotics. Indeed, our mutant did not show increased persistence when exposed to antibiotics of the tetracycline and aminoglycoside family. Furthermore, increased persistence of our *gyrB* mutant was also observed with fluoroquinolones others than ciprofloxacin, but not with older quinolones, such as nalidixic acid and flumequine. This indicates that the hyperpersistence phenotype we selected by repeated exposure to ciprofloxacin is likely specific of compounds that are structurally close to this fluoroquinolone. Sulaiman and Lam (2020) obtained similar results in *E. coli* by demonstrating that tolerance could be specific to the type of antibiotic used during evolution experiment. In summary, our study shows that a *gyrB* mutation, even located outside of the QRDR and conferring a barely significant resistance level, can significantly increase persistence against ciprofloxacin. To our knowledge, it is the first study to report a mutation in a quinolone target gene which confers an hyperpersistence phenotype rather than an increased resistance level. In a recent study, it was also shown that low level quinolone resistance mechanisms mediated by type II topoisomerase modifications increased tolerance and persistence against ciprofloxacin (Ortiz-Padilla et al., 2020). The mechanism involved in this apparently fluoroquinolone-specific hyperpersistence occurring in our mutant remains unknown.

Reconstruction of this *gyrB* mutation in *E. coli* ATCC25922 proved that it was responsible for the hyperpersistent phenotype in this genetic background. However, when reconstructed in two other *E. coli* genetic backgrounds (K12 and B type), this mutation did not confer any hyperpersistence phenotype. This indicates that phenotypic expression of the *gyrB* mutation depends on genetic background and is possibly restricted to a narrow panel of strains. To our knowledge, the GyrB L422P amino acid substitution has not been reported in any *E. coli* clinical or laboratory isolate, or at an equivalent position in another bacterial species. We cannot exclude that its selection was influenced by our *in vitro* experimental design and that its selection would be unlikely *in vivo*. However, it should be noticed that strains bearing such a hyperpersistence-conferring mutation would probably not be remarked in a clinical setting, where antibiotic resistance is the only bacterial phenotype that is measured and considered to make antibiotic therapy decisions. Our study shows that a mutation located outside of the QRDRs of target genes can lead to strong alterations of the bactericidal effect of fluoroquinolones without increasing significantly the resistance level. In a more general manner, it is possible that some mutations detected in antibiotic target genes and which are overlooked because they are not associated with a resistance phenotype, actually confer a tolerance or hyperpersistence phenotype that can compromise treatments.

Knowing the role played by tolerant and persistent bacteria during therapeutic failure, it now seems essential to take these phenotypes into consideration when choosing an antibiotic treatment. Currently, analyses carried out in clinical settings (antibiograms, MIC determinations) allow measurement of susceptibility level but not detection of tolerance and hyperpersistence phenotypes. This can lead to classify bacterial isolates as susceptible whereas they could be difficult to treat and lead to antibiotic treatment failure and infection relapse due to an undetected tolerance or persistence phenotype. Although techniques have been developed, such as Tolerance Disk test (TDtest), which is based on the disk-diffusion assay (Gefen et al., 2017), they remain unreliable and tedious to implement on a large scale. There is a clinical need to develop technical means to detect hyperpersistence and tolerance phenotypes in an automated and rapid way.

## Data Availability Statement

The nucleotide sequence data are deposited in the EMBL-EBI European Nucleotide Archive repository under accession number PRJEB52382.

## Author Contributions

EG contributed to conception and design of the study and supervised this project. AP, QV, CT, and NE performed the experiments. CT and NE designed mutagenesis experiments. AP, QV, and EG analyzed the data. AP wrote the first draft of the manuscript. CT, NE, QV, AB-M, and EG revised the manuscript. All authors contributed to the article and approved the submitted version.

## Conflict of Interest

The authors declare that the research was conducted in the absence of any commercial or financial relationships that could be construed as a potential conflict of interest.

## Publisher’s Note

All claims expressed in this article are solely those of the authors and do not necessarily represent those of their affiliated organizations, or those of the publisher, the editors and the reviewers. Any product that may be evaluated in this article, or claim that may be made by its manufacturer, is not guaranteed or endorsed by the publisher.
