# Supplementary material for: Repeated Exposure of Escherichia coli to High Ciprofloxacin Concentrations Selects gyrB Mutants That Show Fluoroquinolone-Specific Hyperpersistence
Source: Front Microbiol. 2022 May 30;13:908296. doi: 10.3389/fmicb.2022.908296 (PMC9189390; doi:10.3389/fmicb.2022.908296)
Supplement: Supplementary file 1 [file Data_Sheet_1.PDF]

Supplementary data

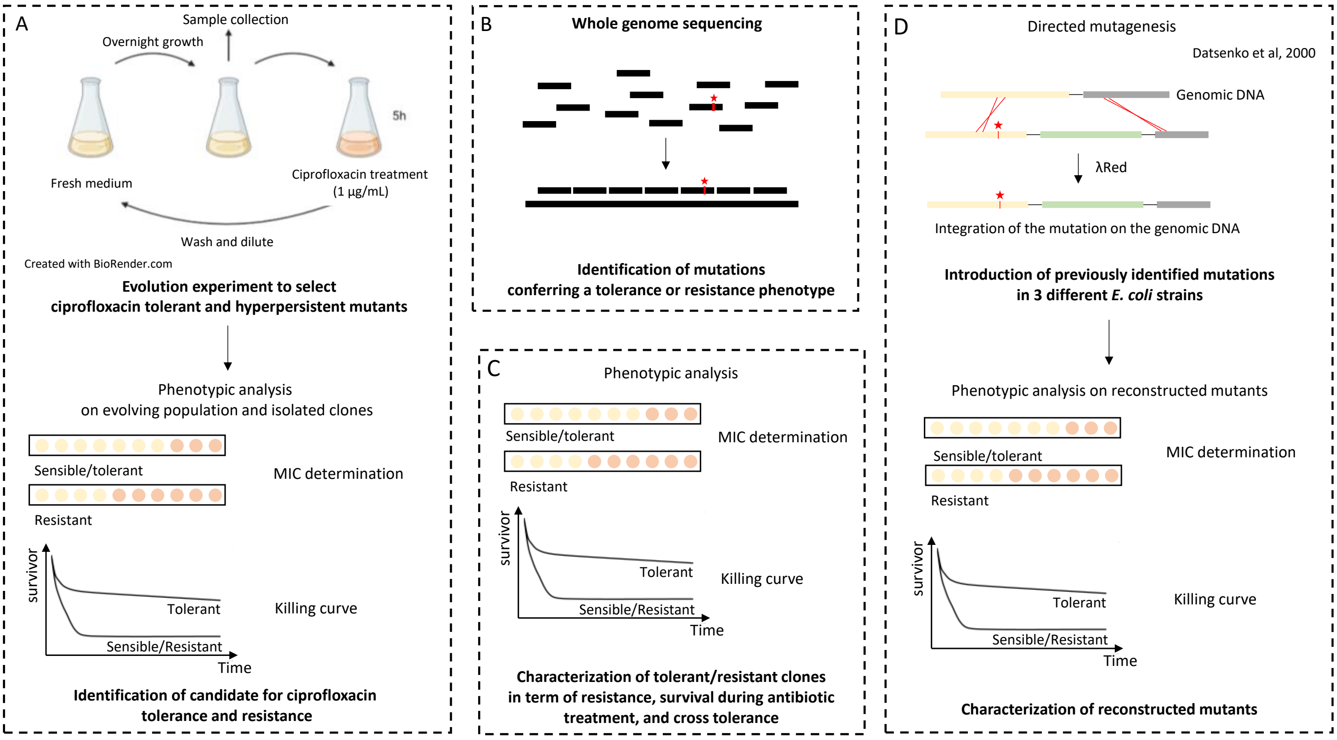

Figure S1*:* Summary diagram of the different steps performed in the study of tolerance to ciprofloxacin in *Escherichia coli* ATCC25922. **(A)** First, a bacterial evolution experiment was set up to select ciprofloxacin tolerant or hyperpersistent mutants. Bacteria were repeatedly exposed to therapeutic concentrations of ciprofloxacin. To identify ciprofloxacin tolerant and resistant candidates, phenotypic tests (MIC determination and bactericidal assays) were performed on the evolving populations and on isolated clones. **(B)** Whole genome sequencing was performed on previously identified clones. Mutations associated to tolerance/hyperpersistent and resistance phenotype were identified. **(C)** Mutants were characterized in terms of resistance, survival upon antibiotic treatment, cross-tolerance to other fluoroquinolones, quinolones, and other antibiotic classes. **(D)** To confirm that the hyperpersistent and resistance phenotypes were due to the identified mutations, these latter were introduced by homologous recombination in 3 different *Escherichia coli* strains: *E. coli* ATCC25922*, E. coli* MG1655 and *E. coli* BL21 after which phenotypic analysis were performed as described above.

Primers	5' – 3' Sequences	Function
MBA 1 forward	CGTCTGCGTGAGTTGTCGTTCCTCAACTCCG GCGTTTCCATTCGTCTGCGCGACAAGCG	Amplification of gyrB DNA sequence on *E. coli* ATCC25922 WT, T and R genome; final amplification
MB2 reverse	AGAGCCGCCCGCGGAGTCCCCTTCCACCAA GTACAGTTCGGAAAGCGCCGGATCGCGTT	Insertion of tolerance mutation on *E. coli Mg1655* and *BL21* recombinant substrate
MB2 forward	AACGCGATCCGGCGCTTTCCGAACTGTACTT GGTGGAAGGGGACTCCGCGGGCGGCTCT	
MB3 reverse	GCGCGGTGATAAGCGTCGCCACTTCCTGAT AAGAGAGCATCTTATCGAAGCGCGCTTTC	Insertion of resistance mutation on *E. coli Mg1655* and *BL21* recombinant substrate
MB3 forward	GAAAGCGCGCTTCGATAAGATGCTCTCTTAT CAGGAAGTGGCGACGCTTATCACCGCGC	
MBA4 reverse	GAAGCAGCTCCAGCCTACACTCGTAGGCCT GATAAGCGTAGCGCATCAGGCACGCTCGC	Amplification + homology sequence for assembly of different PCR fragments
MBA4 forward	GCGAGCGTGCCTGATGCGCTACGCTTATCA GGCCTACGAGTGTAGGCTGGAGCTGCTTC	Amplification of FRT-Cm^{R}-FRT cassette + homology sequence for assembly of different PCR fragments
MB5 reverse	GCCTACAAAATCATGAAAATTCAATACATTG CAAGATTTCATATGAATATCCTCCTTAG	FRT-Cm^{R}-FRT cassette amplification for *E. coli Mg1655* and *BL21*
A5 reverse	GCCTACAAAAACATGCAAATTCAATATATTG CAGGAGTCCATATGAATATCCTCCTTAG	RT-Cm^{R}-FRT cassette amplification for *E. coli ATCC25922*
RecF5	TCTACCGACGACATTATTCAGG	Verification of the introduction of the *gyrB* gene mutations + check excision of the *cat* gene
RecF6	TATCCTGCGTTCAGTAACATCC	Verification of the introduction of the *gyrB* gene mutations + sequencing
RecF14	ATACGATACCAGCAGCACAGC	check the excision of the *cat* gene
RecF13	CGTCGTATGCTGCGCGTTACCG	Sequencing of the genomic region after excision of the *cat* gene

Table S1 : Primers used in site-directed mutagenesis.

MB: primers used for *E. coli* MG1655 and *E. coli* BL21; MBA: primers used for *E. coli* MG1655; *E. coli* BL21 and *E. coli* ATCC2599

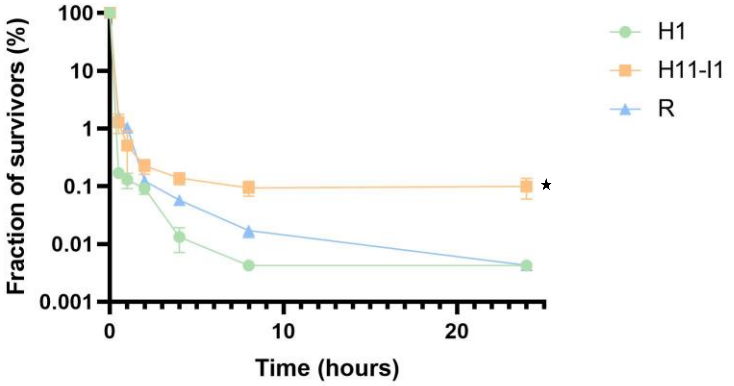

Figure S2 : The hyperpersistence phenotype in presence of ciprofloxacin is not dependent on inoculum size. Bactericidal test in presence of an inoculum of 10^5 bacteria. Bacteria were exposed for 24 hours to a ciprofloxacin concentration of 256-foldthe MIC. The bactericidal test shows an alteration of killing for strain H11-I1 with an increased fraction of persisters. Significant P values compared with H1 strain are indicated (*P ≤ 0.05).

	MDK_{99}			$MDK_{99.99}$			Survival fraction		
	H1	H11-I1	R	H1	H11-I1	R	H1	H11-I1	R
Ciprofloxacin	0.4	16	2.1	4.2	>24	7.6	$2.5x10^{-7}$	$3.8x10^{-3}$	$3.6x10^{-7}$
Enrofloxacin	0.3	0.4	0.5	2.1	24	5.2	$2.7x10^{-7}$	$9.9x10^{-5}$	$3.1x10^{-7}$
Norfloxacin	1	14.5	1.4	3.6	>24	7.8	$8.7x10^{-6}$	$1.8x10^{-3}$	$3.8x10^{-6}$
Nalidixic acid	11	13	8	24	>24	8	$1.3x10^{-4}$	$1.6x10^{-3}$	$2.5x10^{-5}$
Flumequine	4.2	6	5	>24	>24	>24	$1.6x10^{-4}$	$7.9x10^{-4}$	$1.4x10^{-3}$
Tetracycline	2.6	5	2.6	12.5	17.6	12	$1.3x10^{-5}$	$8.1x10^{-6}$	$3.4x10^{-6}$
Gentamicin	0.2	0.2	0.2	0.5	0.5	0.54	$3.0x10^{-7}$	$3.1x10^{-7}$	$3.8x10^{-7}$

Table S2 : Summary table of MDK values and survival fraction representative of tolerance and persistence phenotypes. MDK_{99} and $MDK_{99,99}$ represent the time required to eliminate 99% and 99.99% of the bacterial population respectively. Survival fraction was measured after 24 hours of antibiotic treatment and represents the rate of persisters.

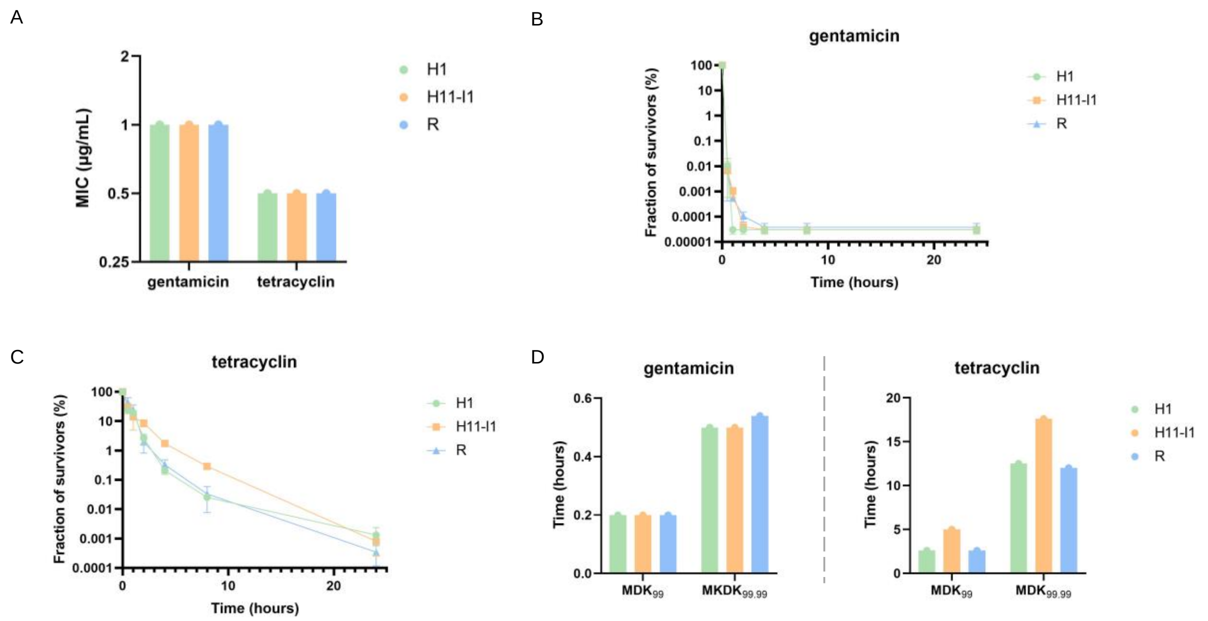

Figure S3 : No cross-persistence is observed with antibiotics of the cyclin and aminoglycoside families. **(A)** MICs of the H1 parental strain, the hyperpersistent clone (H11-I1) and the resistant clone (R) were determined for two different classes of antibiotics: an aminoglycoside (gentamicin) and a cyclin (tetracycline). MICs of the H11-I1 strain were identical to those of the parental strain for all antibiotics tested. **(B, C)** Killing assays with gentamicin and tetracycline showed no significant alteration of bacterial killing for the H11-I1 mutant. **(D)** MDK_{99} and $MDK_{99.99}$ were determined from the killing curves. No significant change in MDK_{99} and $MDK_{99.99}$ was observed. All three strains were killed similarly by gentamicin, whereas a slight increase (x2) in MKD_{99} was observed with tetracycline in the H11-I1 mutant.

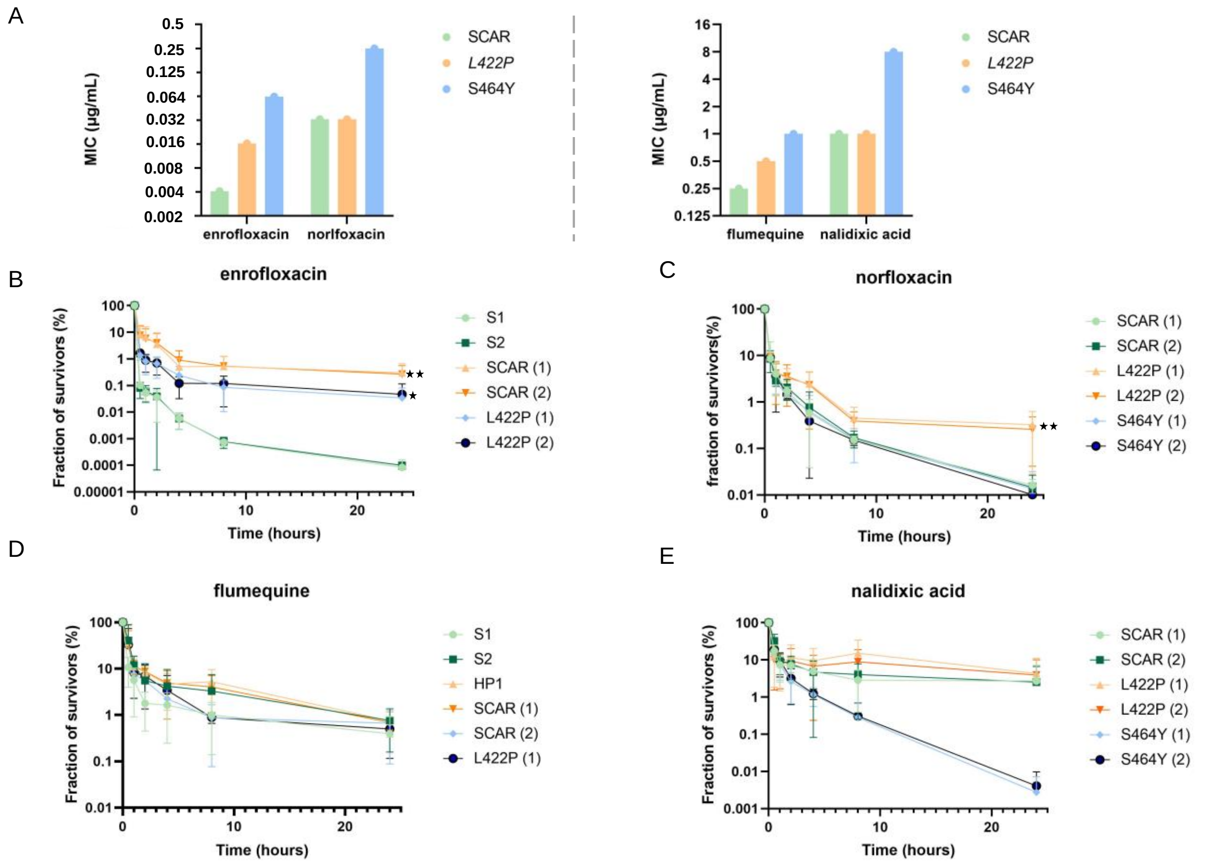

Figure S4 : After reintroduction of mutations in *E. coli* ATCC25922, the mutation L422P is still associated with an hyperpersistent phenotype for fluoroquinolones. **(A)** The MICs of two fluoroquinolones (enrofloxacin and norfloxacin) and two older generation quinolones (nalidixic acid and flumequine) were determined for the different reconstructed susceptible (S), hyperpersistent (HP) and resistant (R) mutants. **(B, C)** Bactericidal tests in presence of fluoroquinolones (enrofloxacin and norfloxacin) show an altered bactericidal effect for the HP clones **(D, E)** Bactericidal assays with old generation quinolones (flumequine and nalidixic acid) show no alteration of bactericidally for the HP clones. Significant P values compared with the H1 strain are noted (*P ≤ 0.05; ** p≤ 0.01)
